# Management of Bone Health in Adult Mastocytosis

**DOI:** 10.1007/s11914-025-00901-w

**Published:** 2025-02-13

**Authors:** Yannick Degboé, Coralie Nezzar, Pauline Alary, Masson Maëva, Cristina Bulai Livideanu, Michel Laroche

**Affiliations:** 1 Rheumatology Department, CHU de Toulouse and Université de Toulouse, Toulouse, France; 2 Mastocytosis Expert Centre – CEREMAST Toulouse, Toulouse, France; 3 INSERM U1291 INFINITY, Toulouse, France; 4 Dermatology Department, CHU de Toulouse, Toulouse, France

**Keywords:** Systemic mastocytosis, Bone, Osteoporosis, Fracture, Treatment, Bisphosphonates

## Abstract

**Purpose of Review:**

The present review will examine bone disease in mastocytosis, analyze the existing literature on its management, and propose a strategy for osteoporosis treatment in systemic mastocytosis. This strategy is based on both the available scientific evidence and the experience gained at our expert center (CEREMAST).

**Recent Findings:**

Systemic mastocytosis is a rare disorder, primarily affecting the bone and leading to osteoporosis, bone pain, and bone structural abnormalities. While traditionally described in indolent systemic mastocytosis, bone involvement is also observed in bone marrow mastocytosis. The true prevalence of systemic mastocytosis is likely underreported, highlighting the importance for clinicians to be familiar with the condition, particularly in cases of osteoporosis. Osteoporosis management typically involves bisphosphonates, with potential benefits from combining them with specific treatments like interferon in severe osteoporosis with vertebral fractures. The potential of new mast cell-targeting molecules to treat bone involvement needs to be demonstrated.

**Summary:**

This review provides a guide for osteoporosis and bone pain management in systemic mastocytosis.

## Introduction

Mastocytosis is a heterogeneous group of rare diseases related to the accumulation/proliferation and activation of pathological clonal mast cells in several organs [1]. Mast cell deregulation is responsible for symptoms related to the release of mast cell mediators and/or to tissue infiltration by mast cells [2]. Notably, the skeleton is a preferential target tissue, especially in the systemic form of mastocytosis, i.e., systemic mastocytosis (SM). Given the rarity of this pathological condition, the management of bone health in mastocytosis largely depends on the opinions of experts [3]. Only a few dedicated studies are available, mostly consisting of case reports and small cohorts, but no randomized controlled trial has been conducted to date. Here, we review the features of bone disease in SM and its management.

## Mastocytosis and Bone Disease

### Types of Bone Involvement

**Fig. 1:**
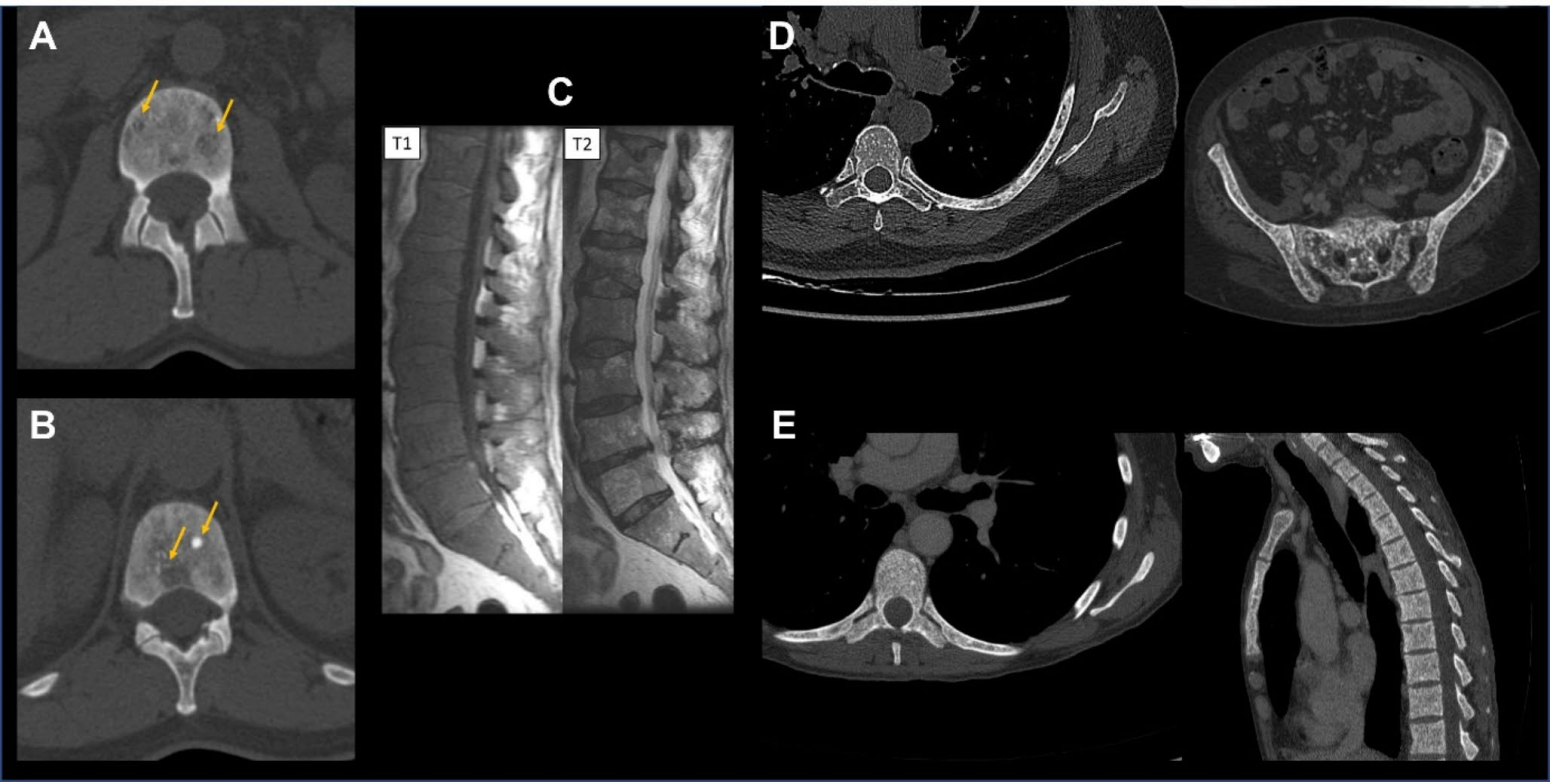
Main bone radiological involvement in systemic mastocytosis. (**A**) Spine CT-scan with vertebral focal osteolytic lesions. (**B**) Spine CT-scan with mixed lesions on the same vertebra with both focal osteolysis and focal osteocondensation. (**C**) Spine MRI with diffuse infiltration consisting of hypointense signal in T1-weighted sequence and heterogeneous T2 signal in fat-saturated sequence. (**D**) Spine and pelvis CT-scan with disseminated punctiform osteocondensations. (**E**) Spine CT-scan with diffuse bone marrow sclerosis/osteosclerosis

Bone involvement in systemic mastocytosis is highly heterogeneous. It can include: radiological findings (Fig. 1) such as diffuse bone resorption referred to as osteoporosis; focal bone resorption referred to as osteolysis; diffuse bone condensation referred to as osteosclerosis; focal bone condensation; and bone marrow infiltrate [4, 5]. Mixed patterns are common. Thorough analysis of bone lesions is important, as they serve as prognostic markers. Large osteolytic lesions (i.e., > 2 cm), diffuse osteosclerosis and bone marrow infiltrate (hypointense T1W, hyperintense T2W and STIR signal in MRI) are patterns observed in advanced SM, whereas osteoporosis, small osteolytic lesions and focal bone condensation are usually observed in non-advanced SM [6].

In addition, patients often report bone pain, with this non-specific manifestation occurring in 10 to 31% of cases [7–9]. This assessment remains a challenge since it requires medical expertise in musculoskeletal pathologies (often by the rheumatologist) to rule out differential and/or associated diagnoses, including common conditions such as osteoarthritis, common low back pain and fibromyalgia.

### Pathophysiology of Osteoporosis in Mastocytosis

Two histomorphometry studies have described the bone remodeling abnormalities in SM [10, 11]. SM is associated with a moderate bone hyper-resorption, characterized by an increase in the number and activity of osteoclasts [10, 11]. The resorption lacunae are not in direct contact with the mast cell nodules [10]. In parallel, osteoblastic formation is similar to that of osteoporotic patients without mastocytosis, with mast cells adjacent to osteoblasts or lining cells. VCAM-1 adhesion protein expressed by mast cells is thought to enable their adhesion onto osteoblasts/lining cells expressing α4β7 or α4β1 integrins [10]. There are also mineralization abnormalities, with a significant increase in the bone formation rate (BFR/BV ratio) [10]. Mast cells are thought to influence bone cells through the secretion of heparin, histamine, tryptase, IL-1, and IL-6 [12–15]. In addition, a more recent study has identified micro-RNA (miR-23a and miR-30a) contained in mast-cell-derived extracellular vesicles as potential new players of this pathogeny [16].

### Epidemiological Considerations for Osteoporosis

Among all forms of mastocytosis, indolent SM (ISM) has historically been considered as associated with bone involvement and osteoporosis [17, 18]. SM prevalence in Europe is estimated at 1/1000 to 1/10,000. However, this value is probably largely underestimated due to the non-specific nature of its symptoms and a lack of awareness of the disease on the part of both practitioners and the general public. Single-center data from a German study showed that with systematic bone biopsy in 1374 patients referred for osteoporosis, the prevalence of indolent SM was 0.5%, and even > 5% in young men with osteoporosis [19].

Osteoporosis is a classical manifestation of SM, affecting up to 30% of patients, including young males (who are not commonly affected by osteoporosis), and it is responsible for a specific spine osteoporosis and multiple vertebral fractures [7, 20–25].

Among SM forms, bone marrow mastocytosis (BMM) is now individualized from ISM, and is characterized by the absence of skin lesions and B-findings and a basal serum tryptase below 125 ng/ml [26, 27]. BMM is frequently observed in rheumatological practice and does not differ from ISM in terms of bone presentation. In our expert center, BMM represents ~ 17% of all cases of non-advanced systemic mastocytosis (including smoldering systemic mastocytosis) [21].

Other forms of mast cell diseases may be associated with bone involvement and especially osteoporosis. Recent data highlight the high frequency of osteoporosis in monoclonal mast cell activation syndrome (MMAS) [21, 28]. However, its presentation seems to differ from that of ISM and BMM, with frequent additional explanatory risk factors for osteoporosis and fewer vertebral fracture cascades [21].

### Tools to Assess Bone Health

Bone involvement can be readily assessed in routine care. Biological assessment relies on measuring the markers of bone metabolism. These includes (but are not limited to) serum calcium and phosphorus, 25OH vitamin D, alkaline phosphatase, and serum C-telopeptides of collagen type 1 (serum crosslaps or CTX, as biomarker of bone resorption). Radiological assessment involves measuring bone mineral density (BMD) by dual x-ray absorptiometry (DXA), X-rays, CT-scan and MRI. Methods of bone health assessment are recalled in Table 1.

**Table 1:** Routine methods of bone health assessment in mastocytosis. ALP: alkaline phosphatase; DXA: dual x-ray absorptiometry; GFR: glomerular filtration rate; MRI: magnetic resonance imaging; PET-scan: Positron emission tomography; SD: standard deviation; WHO: World Health Organization

Method	Readout
*Bone clinical assessment*	
History of vertebral fractures	Identification of bone fragility (potential osteoporosis)
Bone pain	Identification of bone lesion or bone marrow infiltrate
*Bone biological assessment*	
Calcemia, phosphatemia, 25OH-vitamine D, GFR, ALP	Identification of associated metabolic abnormalities potentially affecting bone health
Serum crosslaps (CTX)	Evaluation of osteoresorption level
*Bone imaging assessment*	
DXA	Identification of osteoporosis or osteosclerosis. Based on WHO criteria, osteoporosis is defined as a T-score ≤-2.5 SD in postmenopausal women and males > 50 years old. In young adults, osteoporosis is defined by a Z-score <-2 SD (non-consensual threshold). Osteosclerosis is defined as a T-score or Z-score > + 2.5 or + 4 (non-consensual threshold).
X-rays	Identification of focal or diffuse bone abnormalities such as demineralization, fracture, condensation/sclerosis in axial skeleton and rarely in appendicular skeleton.
CT-scan	Identification of focal or diffuse bone abnormalities such as demineralization, fracture, condensation/sclerosis in axial skeleton and rarely in appendicular skeleton.
MRI	Identification and timing of vertebral fractures (recent and semi-recent fractures appear with bone oedema). Identification of patterns of advanced mastocytosis such as diffuse sclerosis or bone marrow abnormal reconversion.
Scintigraphy and PET-scan	Nuclear medicine is not widely used for this indication, but can be useful to identify recent fracture or suspect bone lesions.

X-ray evaluation of thoraco-lumbar spine should be performed in routine to assess the presence of radiographic fractures. CT-scan is useful as second line, and helps to better identify the bone structural abnormalities (osteocondensation, osteoresorption). MRI should not be systematic. MRI appears relevant in case of back pain to confirm recent vertebral fracture (bone marrow oedema of the fractured vertebra), and in case of suspected advanced systemic mastocytosis to evaluate medullar involvement and possible large osteolytic lesions. Given the performance of MRI, bone scintigraphy as a limited usefulness, and may be used as second line (in case of unavailable MRI) to identify recent fractures. These fractures being associated with vertebral hyperfixation of the radiotracer (usually technetium-99 m). To date, PET-CT is not recommended as a routine exam to assess bone health in SM. Its diagnostic value, its prognostic value, and its incremental value (in addition to X-ray/CT and MRI) need further evaluation for bone disease in SM.

### Assessment of Osteoporosis Risk in Mastocytosis

#### Clinical Assessment of Osteoporosis

Trabecular osteoporosis with multiple vertebral fractures is a major hallmark of SM. Clinicians managing these patients have to identify bone fragility and its risk factors. Bone fragility must be mentioned in case of a history of low-energy fracture, defined by a fracture occurred during a fall from one’s height, while walking and obviously when occurring spontaneously.

Special attention should be paid to vertebral fractures, which in one third of cases are asymptomatic. In this case, a loss of stature of 2 cm compared to the previous measurement or 4 cm compared to the historical size indicate a potential vertebral fracture of fractures and therefore should lead to spinal X-ray imaging.

The evaluation of risk factors for osteoporotic fractures largely relies on the usual screening process proposed for postmenopausal osteoporosis [29, 30]. These include risk of fall, glucocorticoid use, early menopause, low body mass index (< 19), alcoholism and smoking.

#### DXA Screening

DXA scan is essential for the assessment of bone strength (Table 1). This exam identifies patients with low BMD defined as a T-score ≤-2.5 SD in postmenopausal women and males > 50 years old. On the contrary to post-menopausal osteoporosis, the threshold for osteoporosis definition in young adults is not consensual. In young adults, osteoporosis is often defined as a Z-score <-2 SD.

Importantly, the DXA is above the threshold of osteoporosis definition in one third of cases of patients with genuinely osteoporotic fractures. A patient with fragility fractures, but a T-score > -2.5 SD should be considered as osteoporotic in most cases [31]. This discrepancy is commonly observed in all forms of osteoporosis, including steroid-induced and postmenopausal, because some of the parameters of bone strength are not evaluated by DXA.

In addition, the presence of vertebral fractures, of osteoarthritis, of surgical equipment or of any situation that artefactually increases BMD in the region of interest generates an overestimation of BMD and subsequently false negatives for DXA-based osteoporosis screening.

Regarding DXA-screening repetition: a close annual follow-up may be considered for SM patients with osteoporosis, especially in severe osteoporosis (with fractures). However, it appears that for SM patients without osteoporosis at the initial assessment, a re-evaluation with bone densitometry within 2 years may not be necessary if the initial crosslaps levels are low [32].

#### Prediction of Fracture Occurrence

To date, the validated predictors associated with the occurrence of vertebral fractures in SM are: low hip T-score; age at diagnosis; and male gender [24, 31]. The level of bone marrow tryptase is also potentially associated with the osteoporotic fracture phenotype in SM; however, this biomarker needs validation in independent cohorts [31, 33]. The value of crosslaps has also been studied. The association between serum crosslaps and osteoporotic fracture phenotype is still the subject of debate [24, 34].

Surprisingly, current covariates proposed to explain OP in ISM are non-specific for ISM. No biomarker specific for ISM is currently used in common medical practice to stratify OP risk in ISM. Serum IL-6 and IL-1β levels may be of interest, but are not used in daily practice [13–15, 35].

From the perspective of prediction, the MastFx score was proposed in 2014 as a means of distinguishing between patients with a high, intermediate or low osteoporotic fracture risk among patients with ISM [24]. This score considers the presence of five items identified at diagnosis: male sex; serum CTX (Z-score of CTX > + 1 SD); bone mineral density of the hip (T score <-1 SD); absence of urticaria pigmentosa; and alcohol consumption. This score needs validation in independent cohorts.

#### Situations in Which Mastocytosis-Related Osteoporosis is Suspected

The following situations are suggestive of mastocytosis-related osteoporosis:

- Vertebral osteoporosis in young males < 40–50 years old;
- Vertebral osteoporosis with cutaneous or “allergic” signs;
- Vertebral osteoporosis not responding to conventional anti-osteoporotic treatments;
- Vertebral fracture cascade in a subject who is not at risk of osteoporosis.

In these situations, SM needs to be ruled out at diagnosis. A thorough skin examination is needed. A first screening step may consist of measuring serum tryptase and searching for KIT mutations in blood by ASO-qPCR or digital PCR, if available [36, 37].

### Treatment of Osteoporosis

#### Points to Consider Before Treating Mastocytosis Osteoporosis

Before treating mastocytosis osteoporosis, clinicians first need to determine whether SM is the only contributor to osteoporosis. If not, management of (i) the associated conditions for secondary osteoporosis (e.g., primary hyperparathyroidism, glucocorticoid use, alcoholism,) or of (ii) postmenopausal status (which represents the main etiology for osteoporosis) is required. To date, no validated biomarker is available to estimate the contribution of SM in osteoporosis when additional causes are associated. Bone marrow tryptase measurement may be a promising biomarker, but still needs to be validated for use in such contexts [20].

Secondly, the presence of vertebral fractures needs to be considered. This feature indicates a severe osteoporosis (according to the WHO definition [29]) and should lead to a more intensive treatment compared to densitometric osteoporosis (i.e., T-score ≤-2.5 without fragility fracture).

Fall risk must also be considered. Falls are markers of frailty and strongly influence the likelihood of fragility fractures. Even though falls are less critical than in non-vertebral fractures, fall risk assessment still has its uses. This assessment includes identification of: (i) risk conditions (balance disorders, sarcopenia, vision disorders, sedative and/or hypotensive medication, cognitive disorders and vitamin D deficiency); (ii) one or several falls during the past year; and (iii) simple dedicated tests in patients without a history of falls (get-up-and-go test, single-leg stance test, sternal nudge test).

The last point to consider is the patient’s metabolic and hormonal status with respect to bone health: ~1000 mg daily calcium dietary intake and sufficient vitamin D intake to reach 25(OH) vitamin D level ≥ 30 ng/ml are recommended in postmenopausal osteoporosis, and should be in osteoporosis of SM.

#### Efficacy of Anti-Osteoporotic Drugs in Mastocytosis

Bone-modifying agents such as bisphosphonates represent the historical cornerstone for osteoporosis treatment in SM [38]. However, this practice is based on expert recommendation without randomized studies to prove their efficacy in SM [2, 7, 39–41]. The limited amount of published research only studies low numbers of patients and contains discrepancies in terms of treatment regimen and, in some cases, insufficient levels of reported data regarding bone assessment, thus explaining the low level of evidence. Importantly, the severity of osteoporosis (i.e., with fragility fracture), the type of bisphosphonate used and the rate of incident fractures during follow-up, although fundamental elements, are often not reported. In densitometric osteoporosis (without fracture), either oral bisphosphonates or intravenous (iv) bisphosphonates (zoledronic acid and pamidronate) seem to represent a relevant option (Table 2). Conversely, in cases of severe osteoporosis, fracture risk is only partially mitigated by bisphosphonates and oral bisphosphonate are insufficient; iv bisphosphonates or other treatments should be considered [40, 42].

**Table 2:** Anti-osteoporotic therapies in indolent systemic mastocytosis. BMD: bone mineral density; DXA: dual x-ray absorptiometry; ISM: indolent systemic mastocytosis; IFN: interferon; IV: intravenous; n.a.: not available TIW: three times a week

1st author, year [ref]	Onnes, 2020 [40]	Orsolini, 2017 [43]	Rossini, 2014 [41]	Barete, 2010 [7]	Lim, 2005 [39]	Laroche, 2011 [47]	Laroche, 2007 [46]	Weide, 1996 [49]
Design	Observational Retrospective	Observational Prospective	Observational Prospective	Observational Prospective	Observational Retrospective	Observational Prospective	Observational Prospective	Observational Prospective
Evaluated molecule	Bisphosphonates	Denosumab 60 mg/6 months	Yearly zoledronic acid 5 mg/year	IV: pamidronate/3 month Oral: risedronate, alendronate, etidronate	Yearly IV pamidronate followed by alendronate or oral alendronate (1 patient)	TIW interferon alfa-2a TIW + monthly pamidronate for 2 years, followed by quarterly pamidronate alone	TIW interferon alfa-2a + monthly pamidronate for 2 years, followed by quarterly pamidronate alone for 2 years	TIW Interferon alpha 2b for 6 to 11 months
Number of patients	58	4	25	23 treated 9 with densitometric follow-up	6	10	4	3
Follow-up duration	7.3 years	1 year	1 year	65 months	0.5 to 3 years	60 months (24 to 102 months)	4 years	9 to 24 months
History of osteoporotic fractures	18/58 (31.0%)	4/4 (100.0%)	13/25 (52.0%)	10/23 (43.5%)	4/6 (66.7%)	10/10 (100%)	4/4 (100%)	2/3 (66%)
Baseline lumbar T-score	-2.7	-2.5 to -3.6	-2.7 +/- 0.7	n.a.	-3.0 (-0.8 to -4.5)	-3.0 +/- 1.0	-2.9	n.a.
Baseline hip T-score	-1.3	-0.6 to -3.0	-1.2 +/- 0.7	n.a.	-1.9 (0.2 to -3.5)	-1.9 +/- 0.7	-2.1	n.a.
Main outcome	*14/58 patients had new fractures after initiation of bisphosphonates therapy * Fracture-free rate: 81.9% at 5 years and 67.0% at 10 years	*1 year increase in spine and hip BMD	* 1 year increase in spine BMD (+ 6%+/- 4.4%) * 1 year increased in hip BMD (2.4% +/- 3.2%)	* Increased spine BMD (+ 11.07% corresponding to 2.05% per year)	* 50%: increase in spine BMD * 50%: stabilization in spine BMD	* 1-year increase in spine BMD: 10.5%+/-6.8 * 1-year increase in hip BMD: 1.6% +/-3.3	* >15% increase in spine BMD over 2 years of IFN + pamidronate combination * Stabilization of BMD with pamidronate alone	* Significant increase in BMD over 1 year (assessed by QCT)
Secondary outcomes	* Increase in spine Z-score over 5 years from − 2.2 to -1.5 * Increase in hip T-score from − 0.9 to -0.5	* Strong suppression of bone turnover markers	* No new fracture over 1 year * Reduction in bone turnover markers	* No new fracture during follow-up * Stable hip BMD	* No new fracture during follow-up * Improvement of bone pain in all patients with bisphosphonates	* 2-year increase in spine BMD: 12.6%+/-5.6 * 2-year increase in hip BMD: 1.9% +/-3.0 * 2-year increase in spine BMD with pamidronate monotherapy: 2.4% +/-0.1 * No new fracture during follow-up	* No new fracture during follow-up	* Reduction in medullar mast cell infiltrate
Main findings	* Significant increase in the 5-year BMD with bisphosphonates (30 patients) * However, new fractures	Increase in BMD at 1-year	Increase in BMD at 1-year	Increase in BMD over 3 years	Possible positive effect of annual pamidronate on BMD	* Major increase in BMD * No additional fracture over 5 years * Higher increase of BMD with combination compared to pamidronate monotherapy	* Major increase in BMD with treatment combination * No additional fracture over 4 years	* Transient increase in BMD, with BMD decrease and new fractures at IFN withdrawal
Limitations	* No description of bisphosphonates type and therapeutic sequences * No data regarding concurrent treatment of ISM	* Limited number of patients * Short follow-up duration * No data regarding concurrent treatment of ISM	* Short follow-up duration * No data regarding concurrent treatment of ISM	* Limited number of patients * Results are not molecule-specific (global report) * Important heterogeneity in treatment regimen * 37% of the patients with concurrent ISM treatment	* Limited number of patients * Heterogeneity in treatment regimen (up to 20 years of treatment) * No data regarding concurrent treatment of ISM	* Limited number of patients * Frequent IFN withdrawal due to tolerance issue	* Limited number of patients	* Limited number of patients * No data about DXA

Denosumab, a monoclonal antibody against the receptor activator of nuclear factor κ B ligand (RANKL), is a potent antiresorptive agent and is approved for postmenopausal and male osteoporosis with high risk of fractures. This drug has been assessed in SM [43]. Despite promising densitometric results, questions remain about both its efficacy in reducing fractures and its tolerance. Importantly, the biological effect of denosumab is associated with a transient (i.e., limited to drug exposition period) and robust inhibition of bone turnover markers [44]. This inhibition of bone turnover markers goes alongside an inhibition of bone resorption reflected by reduced eroded surface and a gain in BMD [44]. However, denosumab withdrawal is associated with a “rebound phenomenon” characterized by: (i) an increase in bone turnover marker levels usually within the first six months following the cessation of treatment; (ii) a decrease in BMD; and (iii) an unexpected increased risk of (multiple) vertebral fractures [45]. Given the propensity of the SM patients to develop vertebral fractures, this rebound phenomenon may be exacerbated after being treated with denosumab and therefore needs careful evaluation and management. Answers would probably be provided by the results of the DenosuMast study (NCT03401060), which evaluates the densitometric (BMD) efficacy of 60 mg of denosumab every six months versus placebo over three years in patients with SM.

Teriparatide, a bone anabolic recombinant peptide derived from intact human parathyroid hormone, is validated for osteoporosis treatment. This drug is of particular interest in patients with predominant vertebral osteoporosis and vertebral fractures. However, to our knowledge, there is currently no probative report of its anti-fracture efficacy in SM. In addition, in our expert center experience, some patients with severe osteoporosis were referred because of teriparatide inefficacy leading finally to the diagnosis of SM [42]. Its place in the management of osteoporosis in SM needs thorough assessment.

#### Efficacy of Mastocytosis Treatment on Osteoporosis

Whereas first line treatment of osteoporosis is usually bisphosphonates, the usual approach to treat secondary osteoporosis is to treat the underlying condition, so as to improve bone strength. As such, interferon, a treatment for SM has been historically proposed to treat osteoporosis in SM. In severe osteoporosis, SM-specific treatments should be considered (Table 2). Here too, supporting studies are limited to case series and small cohorts [42, 46–49]. Importantly, only one study specifically evaluated this challenging situation and reported a positive long-term impact of the combination of interferon (IFN) and pamidronate, with a very high increase in BMD and a low re-fracture rate [47]. Other molecules, such as midostaurin and masitinib, did not demonstrate such efficacy [42].

To date, combining the transient specific effect of IFN with the long-lasting effect of pamidronate seems to be the strategy with the best level of evidence in cases of severe osteoporosis [47]. However, IFN tolerance is often poor. There is no available evidence about mast cell blockers, such as antihistamine, in treating osteoporosis in SM.

#### Proposal for Osteoporosis Treatment

Based on the available data and on our expert center experience, we propose the following strategy for the treatment of osteoporosis in SM.

To treat densitometric osteoporosis (T-score ≤ -2.5 SD without fracture), the first-line treatment involves bisphosphonates. Options include yearly IV (intravenous) zoledronic acid (5 mg), weekly alendronate, or weekly risedronate. For second-line treatment, denosumab or switching between different bisphosphonates should be considered.

In the case of severe osteoporosis with vertebral fractures, the first-line treatment consists of intensive therapy during the first year, including monthly IV pamidronate, potentially combined with weekly peginterferon alfa-2a, followed by quarterly IV pamidronate for another year. Peginterferon alfa-2a should be considered if there are multiple vertebral fractures. If there is intolerance to either interferon or pamidronate, dose reduction may be considered. For second-line treatment, options include quarterly IV zoledronic acid (4 mg) and specific treatments for mastocytosis, such as a new tyrosine kinase inhibitor (ITK). However, the evidence for these alternatives is currently limited.

### Treatment of Bone Pain

#### Ruling Out Differential Diagnosis

Osteoporosis management in SM is often successful. Bone pain management, however, remains difficult due to the lack of a validated treatment. When dealing with a patient with bone pain, the critical first step is to identify its origin. The available studies do not tend to report whether differential diagnosis has been appropriately ruled out. This may explain the important variability in the rate of bone pain across studies. In our expert center experience, common diagnoses, such as osteoarthritis, common low back pain or fibromyalgia, are often the cause of bone pain. In most cases, a proper rheumatological/musculoskeletal examination and first-line examinations, such as X-rays and ultrasounds of painful areas, are sufficient to clarify the etiology when SM-independent.

#### Symptomatic Treatments

Bone pain is thought to be partly related to mast cell degranulation [50, 51]. As such, there is a rationale to proposing mast cell blockers, such as antihistamines and oral sodium cromoglicate. The level of evidence for this practice is weak, with one case report (of a woman aged 40 years) showing an improvement of bone pain after six weeks of inhaled sodium cromoglicate in addition to her usual H1 and H2 antihistamines and oral sodium cromoglicate treatment [52].

#### Bone Treatments

Bone modifying agents, i.e., zoledronic acid, pamidronate and denosumab, are recommended to treat various painful bone disease, including bone metastasis or osteoid osteoma [53, 54]. As pain relief is variable, such molecules could be used as a second-line treatment [39].

#### Radiotherapy

In cases of major refractory bone pain related to bone lesions in the context of advanced systemic mastocytosis, some authors propose palliative radiotherapy [55]. However, radiotherapy shall not be considered as a standard therapy for bone pain in SM.

### Perspectives

Newly developed tyrosine kinase inhibitors (ITKs) targeting KIT D816V, namely avapritinib, bezuclastinib and elenestinib, are currently assessed for use in indolent or nonadvanced SM [56–58]. Until recently, bone involvement, especially osteoporosis, was disregarded in the clinical trials for SM. Recent studies tend to report BMD variation and the improvement of bone pain. Recent congress presentations highlight promising results concerning bone health. Such findings need confirmation. New ITKs may represent a future pathway for treating osteoporosis and bone pain in mastocytosis.

As a matter of fact, drug availability and drug cost are major parameters that influence therapeutic choices. Consequently, optimizing the use of available drugs is imperative. An example of that need is the use of zoledronic acid. In treating SM osteoporosis, pamidronate is used at a dose validated for cancer bony metastasis (monthly infusions); zoledronic acid, however, has only been evaluated at a dose validated for postmenopausal osteoporosis (5 mg yearly infusion *versus* 4 mg every three months). This observation suggests that there is room for improvement with the available and cost-effective therapies.

## Conclusion

Osteoporosis and bone pain represent the main issues encountered in terms of bone involvement in mastocytosis. Intravenous bisphosphonates represent the cornerstone of bone health management in mastocytosis. Newly developed drugs provide promising perspectives on how to improve both osteoporosis treatment and the challenging management of bone pain.

## Key References

- Valent, P., et al., Personalized Management Strategies in Mast Cell Disorders: ECNM-AIM User’s Guide for Daily Clinical Practice. J Allergy Clin Immunol Pract, 2022. 10(8): p. 1999–2012 e6.
  - Comprehensive review of management strategies in mast cell disorders.
- Rama, T.A., et al., Bone and Cytokine Markers Associated With Bone Disease in Systemic Mastocytosis. J Allergy Clin Immunol Pract, 2023. 11(5): p. 1536–1547.
  - Comprehensive analysis of cytokine profile associated with various phenotype of bone involvement in SM.
- Kim, D.K., et al., Mastocytosis-derived extracellular vesicles deliver miR-23a and miR-30a into pre-osteoblasts and prevent osteoblastogenesis and bone formation. Nat Commun, 2021. 12(1): p. 2527.
  - Description of another potential mechanism for bone fragility in SM, based on extracellular vesicles derived microRNA.
- Gehlen, M., et al., Osteoporosis Caused by Systemic Mastocytosis: Prevalence in a Cohort of 8392 Patients with Osteoporosis. Calcif Tissue Int, 2021. 109(6): p. 685–695.
  - Determination of the prevalence of ISM in a cohort of patients with osteoporosis; this study highlighted the higher prevalence of ISM in young males.
- Degboe, Y., et al., The Prevalence Of Osteoporosis Is Low in Adult Cutaneous Mastocytosis Patients. J Allergy Clin Immunol Pract, 2024. 12(5): p. 1306–1312.
  - This study highlighted that abnormal clonal bone marrow mast cell presence with clinical repercussions in patients not fulfilling the SM WHO criteria is not associated with osteoporosis and fractures in cutaneous mastocytosis, but is associated in monoclonal mast cell activation syndrome.
- Khoury, J.D., et al., The 5th edition of the World Health Organization Classification of Haematolymphoid Tumours: Myeloid and Histiocytic/Dendritic Neoplasms. Leukemia, 2022. 36(7): p. 1703–1719.
  - Updated WHO classification for mastocytosis.

## Data Availability

No datasets were generated or analysed during the current study.
